# Thirty-Day Daily Comparisons of Kato–Katz and CCA Assays of 45 Egyptian Children in Areas with Very Low Prevalence of *Schistosoma mansoni*

**DOI:** 10.4269/ajtmh.18-0829

**Published:** 2019-01-02

**Authors:** Ayat A. Haggag, Amal Rabiee, Khaled M. Abd Elaziz, Carl H. Campbell, Daniel G. Colley, Reda M. R. Ramzy

**Affiliations:** 1 Ministry of Health and Population, Cairo, Egypt;; 2 Department of Community, Environmental and Occupational Medicine, Faculty of Medicine, Ain Shams University, Cairo, Egypt;; 3 Center for Tropical and Emerging Global Diseases, University of Georgia, Athens, Georgia;; 4 Department of Microbiology, University of Georgia, Athens, Georgia;; 5 National Nutrition Institute, General Organization for Teaching Hospitals and Institutes, Cairo, Egypt

## Abstract

Forty-five *Schistosoma mansoni* egg–negative/circulating cathodic antigen (CCA) low (Trace-1+) positive children in areas of very low prevalence were followed up daily for 30 days. Stool and urine specimens were collected and examined each day from each child. At the midpoint of the study, three egg-positive control persons with light intensity infection were included in the protocol. Stool samples were examined by the Kato–Katz (four slides/stool sample) technique and all *S. mansoni* egg–negative stools were further tested by the “miracidia hatching test” (MHT). Urine samples were examined by the point-of-care CCA assay (POC-CCA). Over 30 days, only one of 1,338 consecutive stool samples from study subjects was *S. mansoni* egg and MHT positive (0.07%). Egg counts fluctuated daily in stools from positive controls and *S. mansoni* miracidia were detected in all but two samples by the MHT. Point-of-care–circulating cathodic antigen bands were scored from G1 to G10 and then translated to standard Trace, 1+, 2+, 3+ banding patterns. In two districts, the POC-CCA assays were Trace or 1+ for both the study children and the positive controls. In the third district, the POC-CCA assays were Trace or 1+ for the study children and 1+ or 2+ for the positive control. We conclude that in areas with extremely low prevalence *S. mansoni* egg–negative and CCA-Trace or 1+ children are unlikely to pose substantial risks to continued transmission of schistosomiasis. In this setting, POC-CCA Trace or 1+ readings are likely to be false positives or perhaps represent low-level single-sex schistosome infections.

## INTRODUCTION

Schistosomiasis is one of the major neglected tropical diseases worldwide, ranking second only to malaria among parasitic diseases in terms of its socioeconomic and public health importance in tropical and subtropical areas. The disease is caused by an infection with blood flukes of *Schistosoma* spp. and is transmitted to humans through transcutaneous penetration by its larval stages following human direct contact with infested water.^1^ By 2016, it was estimated that at least 209 million people will require preventive treatment.^2^ The disease mostly affects poor and rural communities, particularly agricultural and fishing populations.

The last 40 years have seen a notable decrease in the prevalence and morbidity of the disease in countries that were highly endemic in the past, including China, Brazil, and Egypt.^3^ Furthermore, it is now believed that interruption of transmission, that is, “elimination” of schistosomiasis, is possible in certain areas and has already been reached in some countries and territories including Japan,^4^ Morocco,^5^ and Puerto Rico.^6^ In 2012, the 65th World Health Assembly adopted Resolution 65.21, promoting elimination of schistosomiasis where possible.^7^

Large-scale use of preventive chemotherapy (PC), supplemented with other public health measures, has decreased schistosomiasis transmission rates as evinced through reduced environmental contamination by eggs discharged by infected people. The use of validated methods for monitoring and evaluation of schistosomiasis control/elimination programs is crucial. Presently, a single Kato–Katz examination is widely used for epidemiological field surveys of *Schistosoma mansoni* and is recommended by the World Health Organization (WHO) for mapping endemic areas requiring PC, and for monitoring, evaluation, and surveillance of intestinal schistosomiasis control programs.^8^ As control/elimination programs progress, the prevalence and intensity of infection decrease to low levels and the sensitivity of a single Kato–Katz can be very low due to a combination of factors. These include variation in the distribution of eggs within a single stool specimen, day-to-day variations in egg excretion, and random distribution effects.^9^

Recently, a point-of-care (POC) cassette assay for active *S. mansoni* infection, based on the detection of adult worms’ circulating cathodic antigens (CCA) in urine samples, has been developed and evaluated, and is presently commercially available. Studies in African countries and in Brazil have indicated that the POC-CCA rapid test is more sensitive than the Kato–Katz stool examination for mapping and monitoring *S. mansoni* infection prevalence in moderate (10–< 50%) and high (≥ 50%) prevalence areas.^10,11^ In areas of low prevalence, however, the POC-CCA can be positive for persons who are *S. mansoni* egg negative by the Kato–Katz assay.^12,13^

The Kato–Katz-negative/POC-CCA–positive individuals represent a challenge for control programs shifting their goal, in line with WHO strategy,^7^ to elimination (interruption of transmission). Are they truly egg-negative, such that there is no need to continue mass treatment; or do they sporadically pass viable *S. mansoni* eggs, which might be missed in routine surveys based on the examination of single Kato–Katz assay? Or alternatively, in areas of very low prevalence, are the POC-CCA Trace or 1+ bands by the Kato–Katz egg-negative subjects not sufficiently specific to report active *S. mansoni* infection? A study in Brazil^14^ showed that, in a low transmission setting (prevalence < 10%), the POC-CCA test performed better if trace results were considered as negative cases. Thus, there is a debate about this group (Kato–Katz-negative/POC-CCA–positive individuals), and more data are required to determine their possible contribution to the “end game” of elimination.

In the present report, we address the question of how often egg-negative/CCA-positive children pass viable eggs in their stools. In particular, we followed a cohort of 45 schoolchildren for 30 consecutive days. Data of parasitological examination of stool samples and POC-CCA in urine samples are presented and discussed.

## MATERIALS AND METHODS

### Ethics statement.

The Ethics Review Committee of the Faculty of Medicine, Ain Shams University, reviewed and approved the study protocol (FMASU R5 2017). The Institutional Review Board of the University of Georgia (UGA) evaluated the protocol (STUDY00004772) and determined UGA personnel to be not engaged. The children were enrolled in the study after obtaining informed consent from their parents/guardians. The study objectives and need to obtain daily stool and urine samples were explained to the children and their parents/guardians. The work included only noninvasive collections of stool and urine specimens. Providing a stool and urine sample was taken as a child’s assent.

### Study design and subjects.

**Figure 1.**
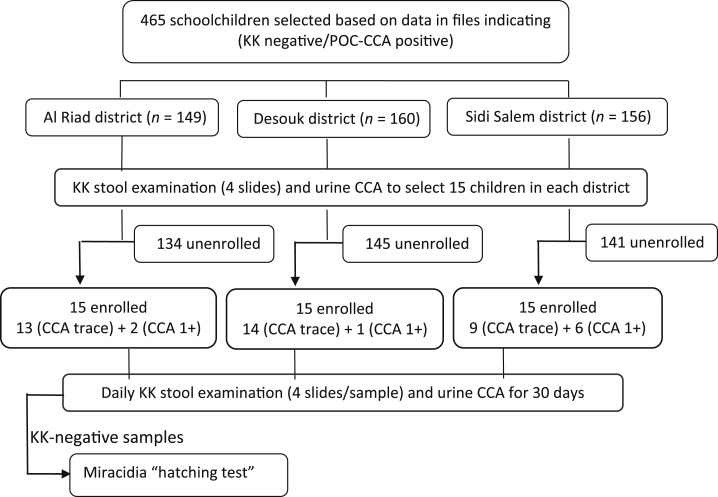
Flow diagram of the subject selection process and study protocol based on stool and urine assays.

The study design is shown in Figure 1. The study was conducted during November and December 2017 and included a cohort of 45 schoolchildren attending schools in three districts (Al Riad, Desouk, and Sidi Salem) in Kafr El Sheikh Governorate, Egypt. Participants were selected from a sample of schoolchildren based on data in files, indicating them to be Kato–Katz *S. mansoni* egg negative/CCA positive. The data in these files were obtained by a previously reported survey in five governorates in the Nile Delta.^13^ The last annual schistosomiasis mass treatment, before the present study, was implemented during the 2014–2015 scholastic year. For the present study, these schoolchildren were reexamined by Kato–Katz (one stool/four slides) and POC-CCA (one urine/one POC-CCA) to select a study cohort of 15 schoolchildren in each district. According to the study protocol, all 15 children had to be egg negative, whereas at least 10 of these subjects should be POC-CCA positive at the Trace level, the other five could be scored 1+ or more.

Stool and urine samples were collected from the 45 children in the cohort daily for 30 days. Each morning, stool and urine samples were brought to the corresponding district laboratory. Stool samples were examined by Kato–Katz (four slides) and all *S. mansoni* egg–negative samples by Kato–Katz were examined by the miracidia hatching test (MHT). Urine samples were examined by the POC-CCA test. Because over the first 2 weeks of the study all Kato–Katz and MHTs were negative, we added one person with a light intensity infection (< 100 *S. mansoni* eggs per gram of feces [EPG]) as a positive control subject in each district. Thus, starting on the 14th–15th day of the study, 15 children and one control subject were followed up per district.

### Kato–Katz thick smear technique.

The Kato–Katz stool analysis was performed according to the WHO standard procedure.^15^ Four slides (41.7 mg each) were prepared and examined from each collected stool sample. The number of detected *S. mansoni* eggs was counted per slide, the number multiplied by 24 and the arithmetic means of the four slides expressed as EPG of stool.

### Point-of-care–circulating cathodic antigen assay.

The POC-CCA test (batch number: 170622073, Exp: 6/2019) was performed according to the manufacturer’s instruction (Rapid Medical Diagnostics, Pretoria, South Africa). Briefly, two drops of urine were added to the sample well of the cassette and allowed to absorb completely into the specimen pad. The test was read after 20 minutes; any line in the test area was considered positive and the band density was recorded. In reading and scoring the POC-CCA results, we used 10 (G1–G10) “standardized POC-CCA cassettes” graded G1 (negative test) to G10 (strong positive), kindly provided by Dr. Govert Van Dam, Leiden University Medical Center, Leiden, the Netherlands. Table 1 indicates the relationship between the G1–G10 grading scale and the more standard evaluations of Negative, Trace, 1+, 2+, and 3+, which will be used throughout this report. The test was considered invalid if the line was developed after 25 minutes or no control line was developed. The test was read and agreed on by two observers (laboratory assistant or laboratory technician), and in case of disagreement, results were discussed with a senior laboratory technician.

**Table 1:** Relationship between the G grading scale and the standard scale of semi-quantifying the band density of the POC-CCA assay

G grading scale	Standard scale equivalent
G1	Negative
G2	Trace
G3	Trace
G4	1+
G5	1+
G6	2+
G7	2+
G8	3+
G9	3+
G10	3+

POC-CCA = point-of-care–circulating cathodic antigen.

### Miracidia hatching test.

In performing this technique, we followed the method described by Lotfy.^16^ From each stool sample, a portion of about 3 gm was emulsified in 75 mL of physiological saline and sieved rapidly through five successive standard brass sieves (mesh openings 2,000, 500, 212, 125, and 32 μm, respectively) by spraying dechlorinated water from a clean 2-L plastic spray bottle (commonly used for spraying agricultural insecticides). Any material that remained on the upper surface of the last sieve (mesh opening 35 μm) was washed with a small amount of dechlorinated water into a 1-L clear conical flask. The flask was covered completely with a black cloth, except the small neck, and was then completely filled with dechlorinated water (pH 7.4–7.6) till the rim. The flask was left strongly illuminated from one side at room temperature. After 2, 4, and 6 hours, the rim of each flask was examined for swimming miracidia using a handheld lens. Observed miracidia indicated a positive MHT, that is, the presence of viable *S. mansoni* eggs in the stool sample. However, the absence of miracidia indicated a negative MHT, that is, no viable, mature *S. mansoni* eggs in the stool sample assayed.

### Statistical analyses.

Data entry was performed on Microsoft Excel database spreadsheet. Descriptive data analysis was calculated with STATA 10 Program. Proportions were compared by Chi-square test and a *P*-value ≥ 0.05 was considered statistically significant.

## RESULTS

### Selection of study participants.

Of the 465 schoolchildren selected based on data in files indicating negative Kato–Katz stool analysis and POC-CCA–positive tests, 160 were from Desouk district, 149 from Al Riad district, and 156 from Sidi Salem district. There were 269 males and 196 females, and their age ranged from 7 to 16 years (mean age ± SD; 11.5 ± 3.0). There was no gender difference between the three districts, but the mean age of schoolchildren in Desouk district (9.4 years ± 1.4) was significantly lower than the mean age of schoolchildren in the other two districts Al Riad and Sidi Salem (12.7 ± 0.9 and 11 ± 1.1 years, respectively) (*P* = 0.01).

The 465 schoolchildren were reexamined using Kato–Katz (one stool, four Kato–Katz slides) and POC-CCA (one test) to select 15 participants in each district to be enrolled in the study. All children were Kato–Katz *S. mansoni* egg negative, except three children who were egg positive (one in Al Riad district and two in Sidi Salem district), and according to the study protocol, they were not enrolled in the study. The age range of the 15 schoolchildren selected in Desouk district was 8–11 years, mean age 9.7 years (±1.0), significantly lower than that of those selected in Al Riad and Sidi Salem districts (*P* = 0.01). The age range of schoolchildren selected in Al Riad and Sidi Salem districts was 10–15 years, mean 12.4 (±0.6), and 9–13 years, mean 11.0 (±1.0), respectively.

In the selection of the study cohort, the POC-CCA reaction was scored in comparison to the control line. Of the 465 schoolchildren, 364 (78.3%) were POC-CCA negative, 87 (18.7%) were Trace, 11 (2.4%) 1+, and 3 (0.6%) > 1+. The POC-CCA–positive reactions in Sidi Salem district were significantly higher than those in the other two districts (*X*^2^ = 13.3; *P* < 0.001). Point-of-care–circulating cathodic antigen detailed data for egg-negative schoolchildren studied in the three districts from which the cohorts were selected are shown in Table 2. Consequently, a cohort of 15 schoolchildren was selected in each district (Figure 1).

**Table 2:** Comparison between the POC-CCA scores of egg-negative children in the three districts from which the study cohorts were selected

District name	Number examined	POC-CCA (Negative) No. (%)	POC-CCA (Trace) No. (%)	POC-CCA (1+) No. (%)	POC-CCA (2+) No. (%)
Desouk	160	134 (83.8)	25 (15.6)	1 (0.6)	0
Al Riad	149	122 (81.9)	25 (16.8)	2 (1.3)	0
Sidy Salem*	156	106 (67.9)	39 (25.0)	8 (5.2)	3 (1.9)
Total	465	364 (78.3%)	87 (18.7)	11 (2.4)	3 (0.6)

POC-CCA = point-of-care–circulating cathodic antigen.

* POC-CCA–positives scores in Sidi Salem district were significantly higher than those in the other two districts (*X*^2^ = 13.3; *P* < 0.001).

### Follow-up of study cohorts.

Enrolled schoolchildren (15 in each district) were followed up for stool and urine collections for 30 consecutive days, except for four participants (see in the following paragraph). In Desouk district, nine of the 15 study children were females. All 15 children were followed up for 30 days. All stool samples examined by Kato–Katz (four Kato–Katz slides/stool sample) were *S. mansoni* egg negative and no miracidia were detected in the MHT. Urine samples were tested by the POC-CCA test and results fluctuated between Negative and 1+ (Table 3). In Al Riad district, 13 of the 15 study participants were females. All 15 children were followed up for 30 days, except ID = 11, for 29 days; ID = 31, for 26 days; ID = 49, for 25 days; and ID = 73, for 28 days. All but one of the stool samples (ID = 120) was *S. mansoni* egg negative by four Kato–Katz slides/stool sample and only the single stool sample from child ID = 120 was MHT positive. All urine samples from Al Riad district were POC-CCA Trace or 1+ (Table 4). In Sidi Salem district, nine of the study children were males. All stool samples examined by Kato–Katz were *S. mansoni* egg negative and all MHT assays negative. All urine samples from the cohort in Sidi Salem district were Trace or 1+ by the POC-CCA assay (Table 5). Therefore, of the 1,338 stools examined over the 30 consecutive days, only one (0.07%) was seen to be positive by Kato–Katz and MHT. Tables 3–5 show that most (89.1%) of the CCA readings of Trace or 1+, over the 30 days of the study period, did not change to Negative from the Trace and 1+ readings on which they were selected for the study.

**Table 3:** POC-CCA scores of 15 study schoolchildren and one adult positive control followed up in Desouk district

Serial No.	ID code	Age (years)	Gender	POC-CCA score*		
				When selected	When followed up	
					Negative (No. [%])	Trace (No. [%])
1	54	11	M	Trace	5 (16.7)	25 (83.3)
2	56	11	F	Trace	8 (26.7)	22 (73.3)
3	61	11	F	Trace	3 (10.0)	27 (90.0)
4	74	11	F	Trace	7 (23.3)	23 (76.7)
5	82	11	F	Trace	17 (56.7)	13 (43.3)
6	96	10	M	Trace	4 (13.3)	26 (86.7)
7	106	10	F	Trace	1 (3.3)	29 (96.7)
8	111	10	F	Trace	11 (36.7)	19 (63.3)
9	116	9	M	Trace	5 (16.7)	25 (83.3)
10	119	9	F	Trace	8 (26.7)	22 (73.3)
11	121	9	F	Trace	8 (26.7)	22 (73.3)
12	124	9	F	Trace	5 (16.7)	25 (83.3)
13	127	9	M	Trace	7 (23.3)	23 (76.7)
14	136	8	M	Trace	6 (20.0)	24 (80.0)
15	146	8	M	Trace	9 (30.0)	21 (70.0)
16	15	35†	M	–	–	13 (100)

POC-CCA = point-of-care–circulating cathodic antigen.

* All 15 study schoolchildren were followed up for 30 days. They were *Schistosoma mansoni* egg negative by four Kato–Katz slides/stool sample per day, and miracidial hatching test negative throughout the 30 days. They were all CCA Trace when selected and fluctuated between Negative and Trace during the 30 days, as indicated by the number and percentage of times Negative or Trace.

† Because of time constraint, a Kato–Katz *S. mansoni* egg–positive control subject of 35 years old was accepted (see details in Table 6).

**Table 4:** POC-CCA scores of 15 study schoolchildren and one positive control child followed up in Al Riad district

Serial No.	ID code	Age (years)	Gender	POC-CCA score*		
				When selected	When followed up	
					Trace (No. [%])	1+ (No. [%])
1	11	13	M	1+	27 (93.1)	2 (6.9)
2	24	13	F	Trace	23 (76.7)	7 (23.3)
3	31	13	F	Trace	25 (96.1)	1 (3.9)
4	38	13	F	Trace	14 (46.7)	16 (53.3)
5	49	13	F	Trace	25 (100)	–
6	73	14	M	Trace	27 (96.4)	1 (3.6)
7	99	12	F	Trace	30 (100)	–
8	103	12	F	Trace	30 (100)	–
9	120	12	F	Trace	29 (96.7)	1 (3.3)
10	124	12	F	Trace	30 (100)	–
11	129	12	F	Trace	30 (100)	–
12	131	12	F	1+	30 (100)	–
13	132	12	F	Trace	29 (96.7)	1 (3.3)
14	133	12	F	Trace	29 (96.7)	1 (3.3)
15	137	12	F	Trace	30 (100)	–
16	16†	11	M	–	13 (76.5)	4 (23.5)

POC-CCA = point-of-care–circulating cathodic antigen.

* All schoolchildren were followed up for 30 days, except ID = 11, for 29 days; ID = 31, for 26 days; ID49, for 25 days; and ID73, for 28 days. All 15 study children were CCA Trace when selected and fluctuated between Negative and Trace during the 30 days, as indicated by the number and percentage of times Negative or Trace. All were *Schistosoma mansoni* egg negative by four Kato–Katz slides/stool sample per day, and miracidial hatching test negative throughout the 30 days, except for ID = 120. On 1 day, on one Kato-Katz slide, one *S. mansoni* egg was found in the stool of ID = 120 and two miracidia were seen from that stool specimen.

† Egg-positive control subject.

**Table 5:** POC-CCA scores of 15 study schoolchildren and one positive control child followed up in Sidi Salem district

Serial No.	ID code	Age (years)	Gender	POC-CCA*			
				When selected	When followed up		
					Negative (No. [%])	Trace (No. [%])	1+ (No. [%])
1	3	13	M	Trace	9 (30.0)	21 (70.0)	–
2	18	11	M	1+	11 (36.7)	19 (63.3)	–
3	19	11	M	1+	2 (6.7)	28 (93.3)	–
4	20	11	M	1+	–	24 (80.0)	6 (20.0)
5	40	11	F	1+	3 (10.0)	21 (70.0)	6 (20.0)
6	46	12	F	Trace	3 (10.0)	24 (80.0)	3 (10.0)
7	47	12	F	Trace	–	30 (100)	–
8	55	11	F	1+	–	26 (86.7)	4 (13.3)
9	56	11	M	1+	3 (10.0)	26 (86.7)	1 (3.3)
10	63	12	M	Trace	2 (6.6)	28 (93.3)	–
11	84	12	F	Trace	2 (6.7)	24 (80.0)	4 (13.3)
12	109	10	M	1+	1 (3.3)	27 (76.7)	2 (6.7)
13	118	10	M	Trace	3 (10.0)	27 (56.7)	–
14	135	10	M	Trace	1 (3.3)	29 (96.7)	–
15	146	9	F	1+	3 (10.0)	26 (86.7)	1 (3.3)
16	17†	10	M	–	–	–	1 (5.9)

POC-CCA = point-of-care–circulating cathodic antigen.

* All schoolchildren were followed for 30 days. All 15 study children were CCA Trace or 1+ when selected and fluctuated between Negative, Trace, and 1+ during the 30 days, as indicated by the number and percentage of times Negative, Trace, or 1+. All were *Schistosoma mansoni* egg negative by 4 Kato–Katz slides/stool sample per day, and miracidial hatching test negative throughout the 30 days.

† Egg-positive control subject. Seventeen times (94.1% of the time) this positive control subject had a CCA score of 2+ (not indicated in the Table).

### Reference positive controls.

Three positive quality control subjects (one in each district) were added and their samples were included in the performance of each assay, starting on the 14th or 15th day of the follow-up. A summary of the data of these positive control subjects is shown by district in Table 6. Note that, when selected, these control subjects had light intensity infection (EPG < 100 *S. mansoni* egg/Kato–Katz slide). In Al Riad and Sidi Salem districts, the control subjects were two males aged 11 and 10 years, respectively. Their Kato–Katz test results fluctuated from negative to light and moderate intensity infections; however, viable miracidia were detected in all MHT assays (Table 6). In Al Riad district, the POC-CCA test results of the positive control ranged between Trace and 1+ (Table 6). In Sidi Salem district, the POC-CCA test results were either 1+ or 2+ (Table 6). In Desouk district, the positive control subject was a 35-year-old male with a light intensity infection, who was included on the 18th day of the follow-up study. His Kato–Katz results fluctuated from negative to light intensity infection (Table 6). Although one *S. mansoni* egg (i.e., 24 EPG), in one Kato–Katz slide, was detected in two successive days, no miracidia were detected in the MHT on these 2 days. Point-of-care–circulating cathodic antigen test results were Trace (Table 6).

**Table 6:** Summary CCA and Kato–Katz data of the three egg-positive control subjects

District	Follow-up days	CCA scores	Kato–Katz slides mean eggs per gram of stool (±SD)				Miracidia hatching test
			1	2	3	4	
Desouk	13*	Trace	11.07 (12.4)	16.6 (20.5)	22.1 (26.7)	29.5 (26.2)	Positive†
Al Riad	16	Trace to 1+	60.7 (63.5)	63.5 (47.2)	60.7 (55.7)	72.0 (56.3)	Positive
Sidi Salem	17	1+ to 2+	76.2 (39.1)	88.9 (40.5)	88.9 (52.1)	70.6 (41.9)	Positive

POC-CCA = point-of-care–circulating cathodic antigen.

* This egg-positive control was added on day 18 of the study because the initial egg-positive control for this district who was used on days 15–17 was seen to be a heavily infected subject (840 eggs per gram). Subsequently, that subject was replaced by a lightly infected subject who was followed up for the remainder of the study (13 days).

† One egg in one Kato–Katz slide was detected on two successive days, but the miracidial hatching tests these 2 days were negative. This is contrary to all other time points, when detection of one egg by Kato–Katz resulted in positive miracidial hatching tests.

## DISCUSSION

This operational research addressed a critical question that needs to be answered if the more sensitive CCA urine assay, rather than the Kato–Katz stool assay, is to be used for routine *S. mansoni* mapping. This is especially true if the CCA urine assay is to be used to guide intervention choices as overall prevalence declines and elimination of *S. mansoni* transmission appears feasible. In this study, we selected and followed a cohort of 45 schoolchildren, 15 each from one of three very low *S. mansoni* prevalence districts (Desouk, Al Riad, and Sidi Salem district), located in Kafr El Sheikh Governorate. The overall *S. mansoni* prevalence in these districts, based on Kato–Katz examination (single slide prepared from one stool sample), was 1.2%, 0%, and 0.9%, respectively.^13^ However, the overall *S. mansoni* prevalence by the POC-CCA was 9.8%, 10.8%, and 7.6% in the same districts, respectively.^13^

Although widely used and useful in many settings, the limitations of the Kato–Katz technique are well documented. It is clear that it lacks diagnostic sensitivity in individuals with low infection intensity or in low endemic areas. Also, the presence of *S. mansoni* eggs in a stool sample varies much more between days than within specimens, indicating that stool sample examination over multiple days is required for accurate prevalence estimates.^17^ Moreover, day-to-day egg count fluctuations in *S. mansoni* infection have been reported, indicating that several examinations on different days may be necessary.^9,18,19^

In the present study, daily stool samples collected over 30 days, from children in two districts (Desouk and Sidy Salem) with CCA Trace or 1+ readings, were uniformly *S. mansoni* egg negative by Kato–Katz (Kato–Katz, one stool/four slides), and no miracidia were detected by the MHT. In parallel, stool samples from positive control subjects, with light intensity infections, showed day-to-day fluctuation in *S. mansoni* egg counts and miracidia were almost always detected by the MHT. In the third district (Al Riad), on 1 day, one study child’s stool was found positive by Kato–Katz and the MHT. This represents one egg-positive stool of 1,338 (0.07%) stools examined in our study of this population of children. Such a finding should be of special programmatic interest, in that it indicates that the population studied in these endemic areas very rarely pass viable eggs in their stools. We propose that such children are likely neither a morbidity threat to themselves nor are they likely to contribute to substantial continued transmission.

The observation that CCA bands in the Kato–Katz *S. mansoni* egg–negative subjects fluctuated between Negative-to-Trace-to-1+ over the 30-day follow-up might be interpreted in two different ways. On the one hand, this could mean that these children still have viable adult worms secreting low levels of CCA antigens. As noted,^13^ these children were previously treated with praziquantel, and if not fully cured or very lightly reinfected, they may have single sex (male) worms or attenuated non-egg-producing female worms.

On the other hand, the specificity of the Trace CCA bands observed for these Kato–Katz-negative children may be lower than that observed in higher prevalence *S. mansoni*–endemic settings. A study, of the evaluation of the POC-CCA in five African countries, reported an average specificity of 72%,^10^ based on a single stool examination. Indeed, the specificity of the POC-CCA in relation to the Kato–Katz stool examination has been addressed in several reports. In Côte d’Ivoire, 14% of POC-CCA–positive individuals were Kato–Katz (duplicate thick smears) negative,^20^ whereas such disagreement reached 54% of children in Uganda.^21^ Furthermore, in a study in Kenya, a day-to-day variation was observed over 5 days of urine collection.^12^ Such data clearly indicate that the POC-CCA should not stand alone as the sole mapping or diagnostic tool for *S. mansoni* infection in areas of low-to-very low prevalence and intensity of *S. mansoni* infections. In addition, the finding that 89.1% of the Trace and 1+ CCA readings did not change to Negative over the 30 days of assays could be seen as supporting an interpretation of these low intensity readings as false-positives in this setting.

In conclusion, the present study provides strong evidence that in areas with very low levels of intestinal schistosomiasis when children are egg-negative (Kato–Katz, one stool/four slides) and CCA Trace or 1+, they are not likely to be a threat to themselves in terms of ongoing egg-focused morbidity or to continued transmission. However, further studies are required to determine if the CCA Trace and 1+ readings from such individuals are altered after one or more treatment cycles with praziquantel.
